# Rapid and simplified post‐processing for simultaneous $B_{0}$ and $B_{1}$ mapping in the application of CEST

**DOI:** 10.1002/mrm.70001

**Published:** 2025-07-22

**Authors:** Mara Quach, Myrte Strik, Rebecca Glarin, Bradford A. Moffat, David K. Wright, Leigh A. Johnston

**Affiliations:** ^1^ Department of Biomedical Engineering and Graeme Clark Institute The University of Melbourne Parkville Victoria Australia; ^2^ Melbourne Brain Centre Imaging Unit The University of Melbourne Parkville Victoria Australia; ^3^ Spinoza Centre for Neuroimaging Amsterdam The Netherlands; ^4^ Department of Computational Cognitive Neuroscience & Neuroimaging Netherlands Institute for Neuroscience, Royal Netherlands Academy of Sciences (KNAW) Amsterdam The Netherlands; ^5^ Department of Neuroscience The School of Translational Medicine, Monash University Melbourne Victoria Australia

**Keywords:** $B_{0}$, $B_{1}$, CEST, inhomogeneities correction, WASABI, WASSR, water shift

## Abstract

**Purpose:**

$B_{0}$ and $B_{1}$ inhomogeneity corrections are crucial for accurate CEST imaging, particularly at ultra‐high‐field MRI. WASABI provides high fidelity δω and $B_{1}$ maps but suffers from prolonged post‐processing and sensitivity to local minima. Our objective was to design an alternative to WASABI's Levenberg‐Marquardt‐based optimization approach to improve both post‐processing speed and accuracy.

**Methods:**

A direct relationship was derived between δω and $B_{1}$ values and information contained in WASABI Z‐spectra. Seven *in vivo* brain datasets were acquired at 7T.

**Results:**

The proposed approach, called RAbi DIstance SearcH (RADISH), accelerated post‐processing by two orders of magnitude, with improved estimation across all brain slices. Maps produced with RADISH were consistent with those produced by unartifacted areas in the original approach, with agreements within 1 Hz and 0.5% for δω and r$B_{1}$ maps, respectively. The percentage of whole‐head artifacts was reduced from 3.90% to 1.05%.

**Conclusions:**

Improvement in speed and robustness provided by RADISH allows for reliable generation of δω and $B_{1}$ maps, contributing to making quantitative CEST imaging at ultra‐high‐field more reliable and advancing its clinical feasibility.

## 1 INTRODUCTION

Chemical exchange saturation transfer (CEST) imaging benefits from improved signal‐to‐noise ratio at ultra‐high field (UHF), enabling increased spectral and spatiotemporal resolutions. For slow‐to‐intermediate exchanging protons (e.g., mobile protein amides, guandinine in creatine, hydroxyl in myoinositol), UHF provides better separation of peaks and in turn can improve Lorentzian‐line‐fit CEST analyses^1^. Additionally, the spill‐over effects are reduced at higher field strengths, beneficial to investigations into species whose resonance frequencies are close to that of water. For the imaging of relatively fast exchanging protons, the effects of UHF are particularly pronounced; one such instance is the amine group in glutamate‐weighted CEST (gluCEST), whose exchange rate with water in physiological conditions has been experimentally reported to be between 5 500 ± 500 Hz^2^ and 7 480 ± 90 Hz^3^ *in vitro*. These rates necessitate a scanner frequency of at least 265 MHz to provide a sufficient chemical shift difference to isolate glutamate‐weighted signal from other solutes, using calculations similar to those by Cember et al. (2023).^4^ However, CEST implementation and reliability at 7T and above are hindered by confounds introduced by increased water shift ($\Delta B_{0}$, often given notation δω), and $B_{1}$ inhomogeneities. Along with normalization and motion correction steps, δω and $B_{1}$ corrections are integral components of standard CEST post‐processing pipelines. As such, δω and $B_{1}$ field maps are typically acquired, the caveat being that as CEST is particularly sensitive to the applied $B_{1}$ field, mapping techniques must provide highly accurate estimates of field inhomogeneities to avoid confounds in the final CEST‐weighted images. Some mapping techniques that have been used to correct CEST images include water saturation shift referencing (WASSR)^5^ and gradient echo mapping^6^ for δω mapping, and double‐^7^/triple^8^‐angle mapping, actual flip angle imaging (AFI),^9^ and Bloch‐Siegert shift (B‐SS)^10^ for $B_{1}$ mapping. More elaborate methods proposed to date include prospective $B_{1}$ homogeneity enforcement techniques using parallel transmission coils for saturation and imaging schemes,^11^, ^12^, ^13^, ^14^, ^15^ or using a dual‐echo readout after CEST preparation to correct for temporal $B_{0}$ drift.^16^ These techniques vary in practicality based on considerations such as SAR limits, hardware availability, and set‐up complexity.

In 2017, Schuenke et al. introduced WASABI (Water Shift And $B_{1}$)^17^ to provide simultaneous estimation of δω and $B_{1}$ maps for correction, achieving results consistent with reference techniques WASSR and B‐SS, respectively. Similar to WASSR, WASABI employs a specialized CEST sequence with transient rectangular saturation pulses to acquire a Z‐spectrum for each voxel that is fitted by a model based on Rabi oscillations to deduce the corresponding $B_{1}$ and δω values. WASABI provides the same advantages as WASSR, namely the user's ability to use the same imaging read‐out and settings as their CEST acquisitions. A variation of WASABI with variable recovery time (delays in‐between offsets) has also been recently proposed, which can simultaneously map $T_{1}$ in addition to δω and $B_{1}$ (“WASAB1T1”).^18^ However, the original technique's dependency on non‐linear least squares fitting using the Levenberg‐Marquardt algorithm (LMA) leads to sensitivity to local minima. Prolonged post‐processing is also an issue, as LMA's dependence on the choice of starting parameters requires an additional step wherein each Z‐spectrum is compared against 37 000 templates before fitting. To date, there is one published alternative^19^ to LMA, which attempts to re‐polarise Z‐spectra and accelerate the template matching process by parameter reduction. However, the re‐polarization step is sensitive to insufficient saturation and/or recovery, and may require additional acquisitions of unsaturated images in between offsets.

Here, we propose a novel post‐processing approach using an algorithm named Rabi Distance Search (RADISH) that is significantly faster and more accurate than the original Levenberg‐Marquardt‐based optimization approach. The original and proposed methods will be referred to as (WASABI &) LMA and (WASABI &) RADISH, respectively.

## 2 METHODS

### 2.1 Theory

Schuenke et al.^17^ introduced the following model to describe Z‐spectra when sampling around the water resonance frequency, with four free parameters: $B_{1}$ (effective $B_{1}$, μT), δω (water shift, Hz), and c and d (approximate surrogates for relaxations and initial magnetization):

(1) $$\begin{array}{ll} Z(\Delta \omega ,\theta ) & =\left|c-d\;\sin^{2}\left(\tan^{-1}\left(\frac{\bar{\gamma }\;B_{1}}{\Delta \omega -\delta \omega }\right)\right)\right.\\ & \;\left.\cdot \;\sin^{2}\left(\pi \;t_{p}\;\sqrt{{\left(\bar{\gamma }\;B_{1}\right)}^{2}+{\left(\Delta \omega -\delta \omega \right)}^{2}}\right)\right| \end{array}$$

where $\theta \in \{c,d,\delta \omega ,B_{1}\}$ is the set of variables to be estimated. Δω is a sampled offset in Hz, $t_{p}$ is the single pulse duration in s, and $\bar{\gamma }$ is the gyromagnetic ratio in $\frac{\text{MHz}}{T}$.

**FIGURE 1:**
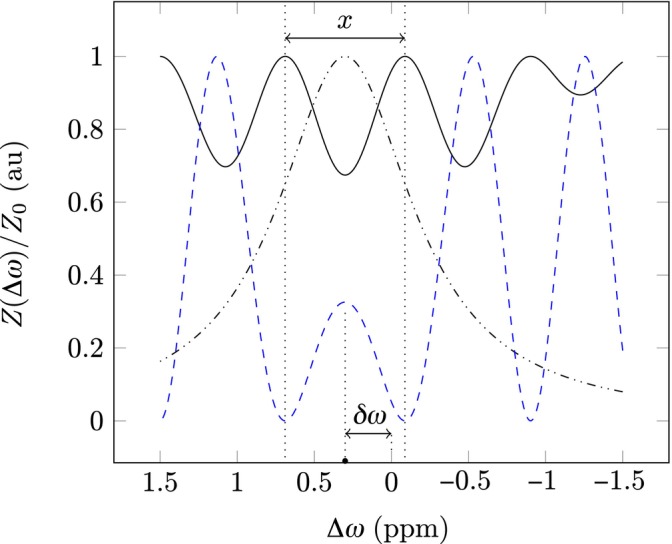
A simulated Z‐spectrum (solid black line) using the WASABI model in Equation (1) with δω=0.3 ppm, $B_{1}=3.7\;\mu T$, c=1, and d=1. x is the distance in ppm between the two local maxima to the left and right adjacent to the axis of symmetry as determined by δω. The first sine‐squared term in the model (1) (black dash‐dotted line) modulates only the amplitude of the Z‐spectrum. The second sine‐squared term in Equation (1) (blue dashed line) modulates the periodicity of the spectrum, reflecting the effects of the saturation power $B_{1}$ on Rabi oscillation in the Z‐spectrum.

The first sine‐squared term in Equation (1) (Figure 1, black dash‐dotted line) modulates the amplitude of the signal and does not contribute to periodicity. The second sine‐squared term in Equation (1) describes the periodic behavior of the Z‐spectrum influenced by Rabi oscillations (Figure 1, blue dashed line). This second term, when considered solely, isolates the signal that is tuned by $B_{1}$, with periodicity unaffected by varying c and d. To account for the effects of δω, we derived a relationship for the distance between the two maxima immediately adjacent to δω (x in Figure 1), producing the following analytical relationship between characteristics of the Z‐spectra and the absolute $B_{1}$ power, in μT, at each voxel:

(2) $$\begin{array}{ll} B_{1} & =\frac{\omega_{0}}{\bar{\gamma }}\;\sqrt{{\left(\frac{n}{\omega_{0}\;t_{p}}\right)}^{2}-\frac{x^{2}}{4}},\\ \text{where}\;n & =\left\{\begin{array}{l} 1,\;\text{if}\;x\leq \frac{2}{\omega_{0}\;t_{p}}\\ 2,\;\text{otherwise} \end{array}\right. \end{array}$$

In Equation (2), x denotes the distance in parts per million (ppm) between the two aforementioned maxima (Figure 1), and $\omega_{0}$ is the reference frequency in MHz. Using the model's symmetric nature, the estimate of δω is given by the midway point between the two maxima. See Appendix A for the full derivation of Equation (2).

### 2.2 The RADISH algorithm

RADISH can be accessed via https://github.com/maraquach/radish. The steps of RADISH are as follows:

For each voxel, let y be the observed Z‐spectrum data:

(3) $$y=[y(\Delta \omega_{1}),\ldots ,y(\Delta \omega_{N})]$$

where N is the total number of offsets sampled.

**FIGURE 2:**
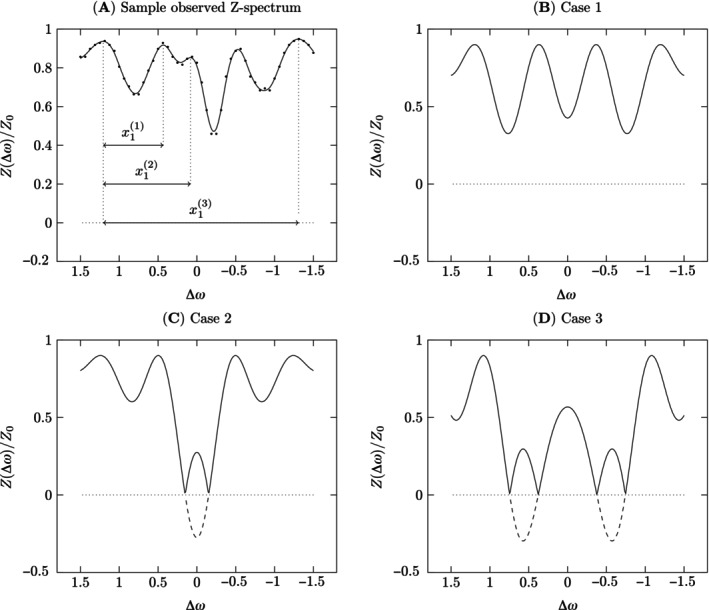
(A) Measured Z‐spectrum (black dots) from a single voxel with asymmetry, fitted by a cubic smoothing spline (solid curve). The amplitude asymmetry, likely caused by a combination of imaging artifacts, noise, and head motion, creates ambiguities in determining the axis of symmetry and confounds the template fitting method used by the original WASABI & LMA technique. $x_{1}^{\left(1\right)}$, $x_{1}^{\left(2\right)}$, and $x_{1}^{\left(3\right)}$ refer to the distance in ppm between the first local maximum identified for the spline and its companion maxima for each case to be considered. (B) Relationship predicted by *Case 1* (Step 3), centered at 0 ppm. This is the simplest case, where $Z(\Delta \omega_{n})>0\;\forall n$. (C) Relationship predicted by *Case 2* (Step 3), centered at 0 ppm, occurring when the Z signal at the axis of symmetry is expected to reach a negative value (dashed line), but is reflected across the *x*‐axis in the magnitude images (solid line). (D) Relationship predicted by *Case 3* (Step 3), centered at 0 ppm, where negative values are expected, but not at the axis of symmetry.

1. Let $\bar{y}$ be the interpolated data after fitting a cubic spline to y with tolerance ϵ.
2. From $\bar{y}$, empirically determine a set of local maxima locations, $ℳ=\{M_{1},\ldots ,M_{m}\}$. Here, m is the total number of local maxima identified for $\bar{y}$.
3. For each $M_{i}\in ℳ$, identify up to three cases of possible δω and x pairs (Figure 2):
*Case 1*: $x_{i}^{\left(1\right)}=|M_{i+1}-M_{i}|$, $\delta \omega_{i}^{\left(1\right)}=M_{i}+\frac{x_{i}^{\left(1\right)}}{2}$, $d_{i}^{\left(1\right)}=1$ for i={1,…,m−1} (Figure 2A)
*Case 2*: $x_{i}^{\left(2\right)}=|M_{i+2}-M_{i}|$, $\delta \omega_{i}^{\left(2\right)}=M_{i}+\frac{x_{i}^{\left(2\right)}}{2}$, $d_{i}^{\left(2\right)}=1.5$ for i={1,…,m−2} (Figure 2B)
*Case 3*: $x_{i}^{\left(3\right)}=|M_{i+4}-M_{i}|$, $\delta \omega_{i}^{\left(3\right)}=M_{i}+\frac{x_{i}^{\left(3\right)}}{2}$, $d_{i}^{\left(3\right)}=2$ for i={1,…,m−4} (Figure 2C)
  Calculate the corresponding $B_{1}$ for each case pair of δω and x according to Equation (2). Each of these cases, j=1,2,3, produces a set of three parameters, $\theta_{i}^{\left(j\right)}=\{\delta \omega_{i}^{\left(j\right)},B_{1i}^{\left(j\right)},d_{i}^{\left(j\right)}\}\in \Theta_{1}$, subject to constraints imposed by exclusion criteria.
4. From Equation (1), using $\theta_{i}^{\left(j\right)}$ with c=1, construct a set of template curves, 𝒵, containing a maximum of non‐zero 3m−7 elements:
𝒵 = $\left\{Z_{1}^{\left(1\right)},\ldots ,Z_{m-4}^{\left(3\right)}\right\}$ ∀i,j from Step 3,
  where $Z_{i}^{\left(j\right)}=[Z(\Delta \omega_{1},\theta_{i}^{\left(j\right)}),\ldots ,Z(\Delta \omega_{N},\theta_{i}^{\left(j\right)})]$.
5. Determine the optimal parameter set, $\theta^{*}=\{\delta \omega^{*},B_{1}^{*},d^{*}\}$:
(4) $$\theta^{*}=\underset{\theta_{i}^{\left(j\right)}\in \Theta_{1}}{\text{arg min}}\|y^{\prime }-{Z^{\prime }}_{i}^{\left(j\right)}{\|}_{1}$$
  where $y^{\prime }$ and ${Z^{\prime }}_{i}^{\left(j\right)}$ are the numerically‐computed first‐order derivatives (central differences) of y and $Z_{i}^{\left(j\right)}$, respectively, and $\Theta_{1}$ is the set of $\left\{\delta \omega ,B_{1},d\right\}$ groups previously found in Step 3 inside their defined boundaries.
6. Increase the precision of the $B_{1}$ value estimation. Equation (4) is optimized for $B_{1}$ using δω and d estimates from Step 5, subject to the constraints that $B_{1}$ lies within the interval $\left[B_{1}(x+l_{1}\times \delta \Delta \omega ),B_{1}(x-l_{2}\times \delta \Delta \omega )\right]$.
7. Repeat for all voxel Z‐spectra to construct the final δω and $B_{1}$ maps.

**FIGURE A1:**
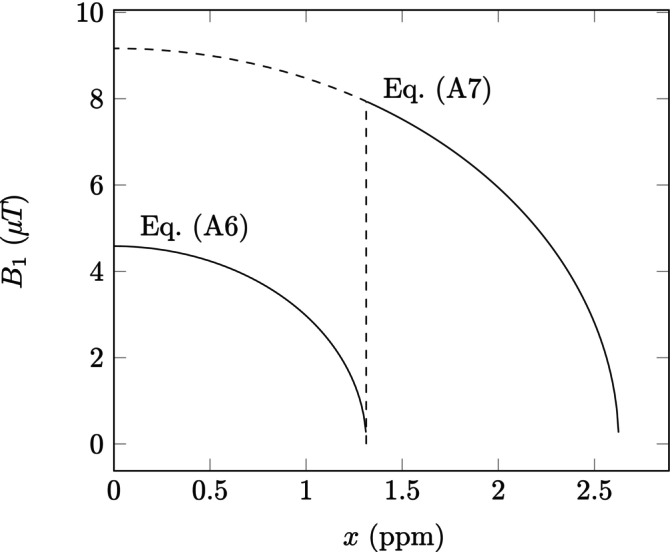
Relationship between $B_{1}$ and the maxima‐pair distance x for n=2,n=4.

In Step 1, fitting the data to a smoothing spline increases SNR in noisy spectra^20^ and minimizes the effects of noise on maxima determination. In Step 3, Case 1 models the simplest scenario where Z(Δω)>0∀n, and *d* = 1. Case 2 models the scenario where the on‐resonant (and nearby) Z‐magnetization is flipped and is depolarized in the magnitude image; *d* is increased to 1.5 to reflect the long $T_{1}$ and/or short recovery time required for this phenomenon to occur. Finally, Case 3 sets *d* to 2 to reflect the instance of an abnormally long $T_{1}$ and high $B_{1}$. For all cases, we assume complete longitudinal recovery between each offset and, therefore, *c* is set to be 1. While theoretically there exists an additional case in which the two maxima of interest are separated by two maxima, our simulations indicate this requires such a strong $B_{1}$ as to be practically unlikely. In Step 5, the use of the derivative as the objective function reduces the number of parameters to optimize to three—as *c* is a linear term (see Supplementary section S1 for more information). In Step 6, the new constraints are dependent on the peak distance instead of $B_{1}$, as a small change in the former can lead to a drastic change in the latter, particularly around x≈1.3 ppm (see Figure A1 for a visualization). $l_{1}$ and $l_{2}$ are adjustment factors to refine x based on the offset step size. The default parameters in RADISH are listed in Table 1, optimized for *in vivo* data acquired at 7T.

**TABLE 1:** Recommended values for RADISH parameters for *in vivo* WASABI data acquired at 7T.

Step	Parameter	Default value
1	ϵ	3e−4
3	$B_{1}$ range	$(0,1.5\times \text{nominal}\;B_{1}]\;\mu T$
	δω range	[−0.6,0.6] ppm
6	$l_{1}$	1.5
	$l_{2}$	2.5

### 2.3 Data acquisition

All imaging experiments were approved by the University of Melbourne Human Ethics Committees. *In vivo* brain scans of seven healthy volunteers (2F, age 24.9 ± 1.9 years) were acquired on an investigational MAGNETOM 7T Plus scanner (Siemens Healthineers, Erlangen, Germany), using an 8Tx‐32Rx head coil (Nova Medical Inc., Wilmington, MA, USA) in circularly‐polarized mode. In addition to WASABI, we acquired WASSR and AFI for comparison δω and $B_{1}$ maps, respectively. Three AFI maps (3M, age 25.7 ± 1.5 years) were ultimately excluded due to aliasing artifacts. Manual whole volume shimming was performed for all subjects to limit water shifts within ± 0.5 ppm, and the same shim settings were applied to all acquisitions for each subject.

The following parameters were used for the WASABI protocol which employed a snapshot 3D‐GRE sequence with rectangular spiral readout^11^: Number of offsets = 49 (−1.5 to 1.5 ppm), nominal $B_{1}$ = 3.7 μT, flip angle = 10°, $n_{p}$ = 1 rectangular pulse, $t_{p}$ = 5 ms, $t_{rec}$ = 5 s, $t_{d}$ = 100 ms, $t_{sat}$ = 5 ms, TR = 7.4 ms, TE = 3.61 ms, matrix = 128 × 128, voxel size = 1.565 × 1.565 × 5 mm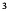, FOV = 200 mm, number of slices = 12. The same saturation and imaging schemes were used for WASSR with these exceptions: Number of offsets = 33, nominal $B_{1}$ = 0.2 μT, $n_{p}$ = 150 Gaussian‐shaped pulses, $t_{p}$ = 25.6 ms, $t_{rec}$ = 3 s, $t_{d}$ = 12 ms, TR = 4 ms, TE = 2 ms. A long saturation train was chosen for WASSR to maximise saturation effectiveness. AFI‐specific parameters were: $t_{p}$ = 300 μs, TR2/TR1 = 5, diffusion damping = 0.7. For each WASABI and WASSR acquisition, an $M_{0}$ image was acquired at Δω=−300 ppm for normalization.

### 2.4 Data processing

All data processing and testing were performed with MATLAB R2023b (MathWorks, Natick, Massachusetts, USA) using in‐house MATLAB scripts, except for the original WASABI method, for which the WASABI authors' original scripts^21^ were used. The default parameters as listed in Table 1 were used in the processing of the data in this study.

WASABI and WASSR images were normalized using the $M_{0}$ image (−300 ppm). To generate reference δω maps, the following objective function based on a reverse Lorentzian line‐fit was minimized voxel‐wise, with $y_{\text{wassr}}$ being the normalized WASSR data:

(5) $$\underset{\delta \omega ,p_{1},p_{2},p_{3}}{\text{arg min}}{\left\|-y_{\text{wassr}}-\frac{p_{1}}{{\left(\Delta \omega -\delta \omega \right)}^{2}+p_{2}}-p_{3}\right\|}_{2}$$

AFI r$B_{1}$ maps were re‐gridded using cubic interpolation to WASABI space. To quantify low‐quality regions in WASABI maps, any δω values exceeding ± 0.15 ppm (approximately two sampling points) deviation from WASSR were categorized to be ill‐estimated. Since r$B_{1}$ is dependent on δω in derivation by both the LMA and our proposed RADISH method, r$B_{1}$ outlier voxels can be assumed to be the same as those in δω maps. To compare maps produced by RADISH and LMA, Bland‐Altman analyses were performed twice: Once on the whole brain maps (12 slices) and once on the middle slices (Slices 5 to 8), both across all subjects.

The performances of RADISH and LMA were also tested with fewer sampling offsets by retrospectively down‐sampling the Z‐spectra by a factor of two (25 offsets).

All statistical analyses were performed with MATLAB and Prism 10 (GraphPad Software, Massachusetts, USA), with significance level α=0.05. Speed evaluation tests were performed with MATLAB using timeit and the Parallel Computing Toolbox on a computer with an 8‐core ARM processor and 16 GB of RAM.

## 3 RESULTS

### 3.1 Qualitative comparisons

**FIGURE 3:**
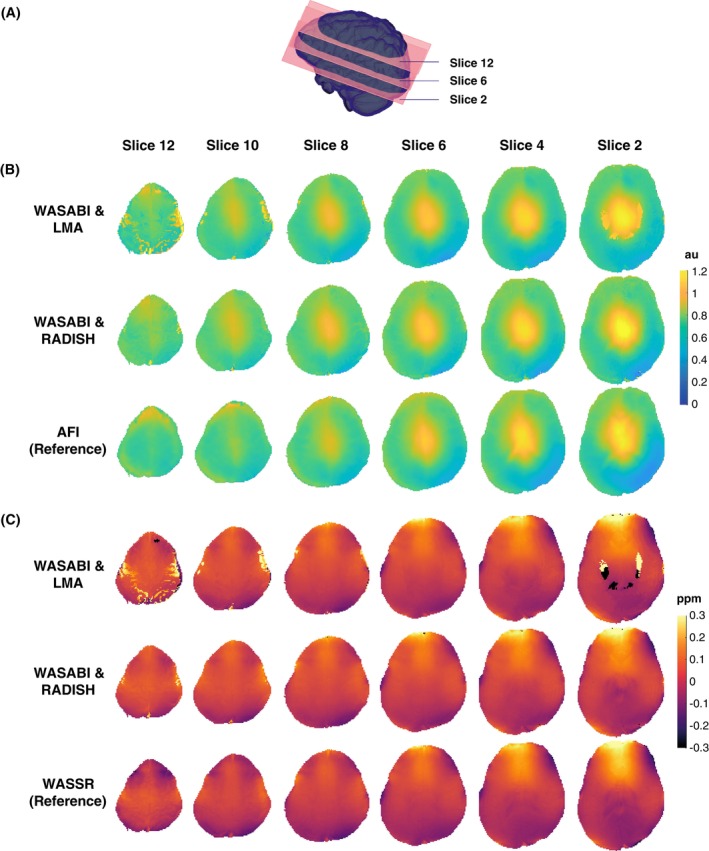
(A) Brain volume demonstrating approximate locations of depicted slices. (B) $B_{1}$ and (C) δω maps generated by WASABI & LMA (top, original method), WASABI & RADISH (middle, proposed), and AFI or WASSR (bottom, the reference technique). Patches of artifact regions are noticeable in LMA maps, particularly in Slices 2 and 12. These artifact regions are consistent between $B_{1}$ and δω maps. Artifacts are observed in RADISH maps, but less frequently. In lower regions (Slice 2), the RADISH δω map is smoother compared to the corresponding LMA map. In AFI maps, some ventricular structures can be observed in Slices 2 and 4, indicating possible confounds due to $T_{1}$ decay effects. Additionally, AFI maps underwent cubic interpolation, resulting in some unreliable regions around the edges of the brain. au: arbitrary units, ppm: parts per million.

Qualitative comparisons between RADISH and the LMA‐based approach can be ascertained using Figure 3 (and Figure S2 in the Supplementary Material). While both techniques produced smooth maps with expected results in middle slices (Figure 3, Slices 4–8), noticeable differences were observed in slices toward the extremes (Figure 3, Slices 2 and 12). In particular, LMA maps exhibited patches of artifact, indicating the algorithm converged to (suboptimal) local minima (e.g., Slices 2, 10, and 12). Only very small areas of artifacts were observed in RADISH maps.

### 3.2 Robustness in comparison to reference techniques

**FIGURE 4:**
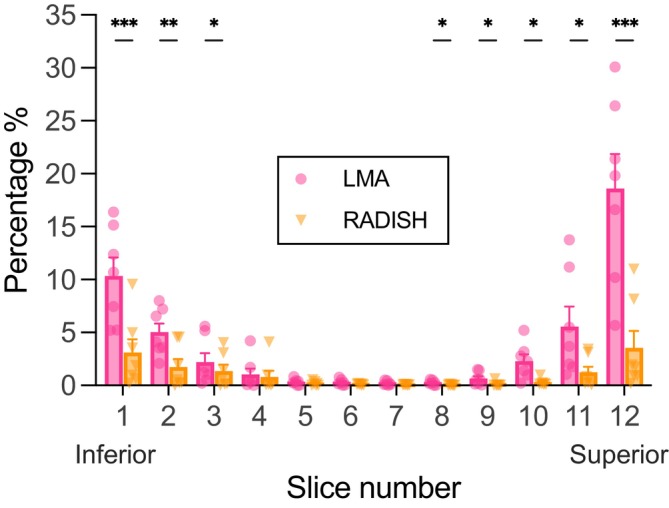
Percentage of ill‐estimated voxels in each slice in maps produced by WASABI & LMA (original) and WASABI & RADISH (proposed) across 7 subjects. Voxels where the calculated δω exceeds ±0.15 ppm (approx. two sample points) deviation from reference WASSR maps were considered to be artifacts. As the estimation of $B_{1}$ is dependent on that of δω, $B_{1}$ artifacted voxels were considered to be the same. *: p<0.05, **: p<0.01, ***: p<0.001.

Figure (4) shows the percentage of ill‐estimated voxels in each slice in maps produced by LMA and RADISH. The mean percentages of ill‐estimated voxels were largest in the most inferior and superior slices for both LMA and RADISH maps (Figure 4). However, RADISH produced fewer artifact voxels in all slices across all 7 subjects. Multiple paired t‐tests revealed that there were significant differences in all except Slices 4–7. The largest mean difference was in Slice 12, with a mean reduction of 15.06% (p=0.0004) by RADISH, while the lowest mean reduction was 0.14% (p=0.20) in Slice 5. Across all slices on average, artifact voxels made up 3.90% of LMA maps, compared to 1.05% of RADISH maps.

**FIGURE 5:**
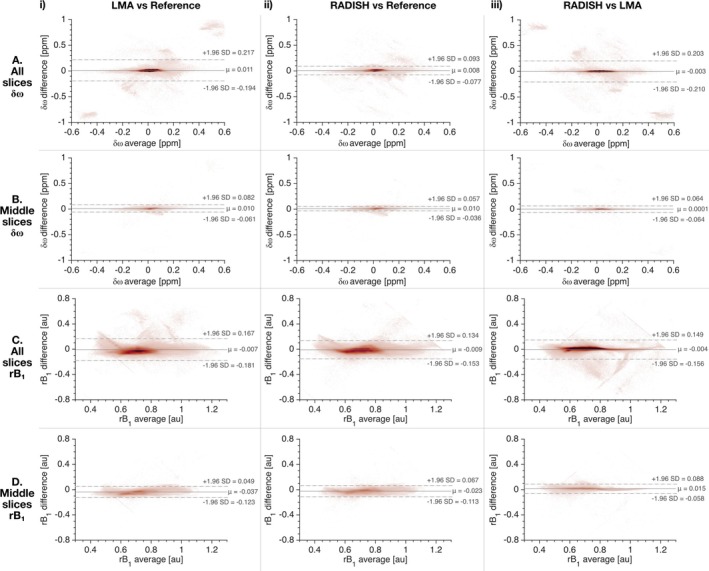
Bland‐Altman plots for LMA, RADISH, and reference maps. (A) Plots for δω analyses of all 12 slices (≈560 000 voxels), with the reference technique being WASSR. These analyses include large areas of artifact voxels in LMA maps, which are visible in plots A.i and A.iii. (B) Plots for δω analyses of only the middle slices (Slices 5 to 8, ≈220 000 voxels), where artifact voxels in LMA maps make up <1% of all voxels. (C) Plots for r$B_{1}$ analyses of all 12 slices, with the reference technique being AFI. Similar to Row A, these include large areas of artifact voxels in LMA maps. (D) Plots for r$B_{1}$ analyses of only the middle slices (Slices 5 to 8), with fewer LMA artifact voxels. With most LMA artifacts removed, the biases between RADISH and LMA maps are low, with narrow limits of agreement for both δω (A.iii) and r$B_{1}$ (C.iii). δω: Water shift, ppm: parts per million, r$B_{1}$: relative $B_{1}$, au: arbitrary unit, μ: Bland‐Altman bias, +/− 1.96 SD: upper/lower limit of agreement.

Results from Bland‐Altman analyses in which reference maps were subtracted from RADISH or LMA maps are shown in Table 1 and Figure 5i,ii. The biases for both LMA and RADISH maps compared to references were low (≈2.4–3.3 Hz for δω and < −1% for r$B_{1}$ maps), indicating good agreements with WASSR and AFI. However, the reduction of artifacted regions by RADISH is evident, resulting in narrower limits of agreement (LoA) for both δω and r$B_{1}$ RADISH maps (Figure 5B,C,E,F).

### 3.3 Comparisons between RADISH and LMA

For all 12 slices, Bland‐Altman analyses showed the bias between LMA and RADISH maps were ≈0.09 Hz for δω at 7T and <1% for r$B_{1}$, indicating a good agreement between the two techniques (Table 1, Figure 5A.iii and C.iii). However, the δω LoA was considerably wider than that between RADISH and WASSR due to artifacts in LMA maps. In the middle slices where the percentage of ill‐estimated LMA voxels was less than 1%, the LoA was narrow, with 90% of voxel‐pair differences falling between −0.07 and 0.07 ppm and −0.058 and 0.088 for δω and r$B_{1}$ maps, respectively.

### 3.4 Effects on final CEST contrast

**FIGURE 6:**
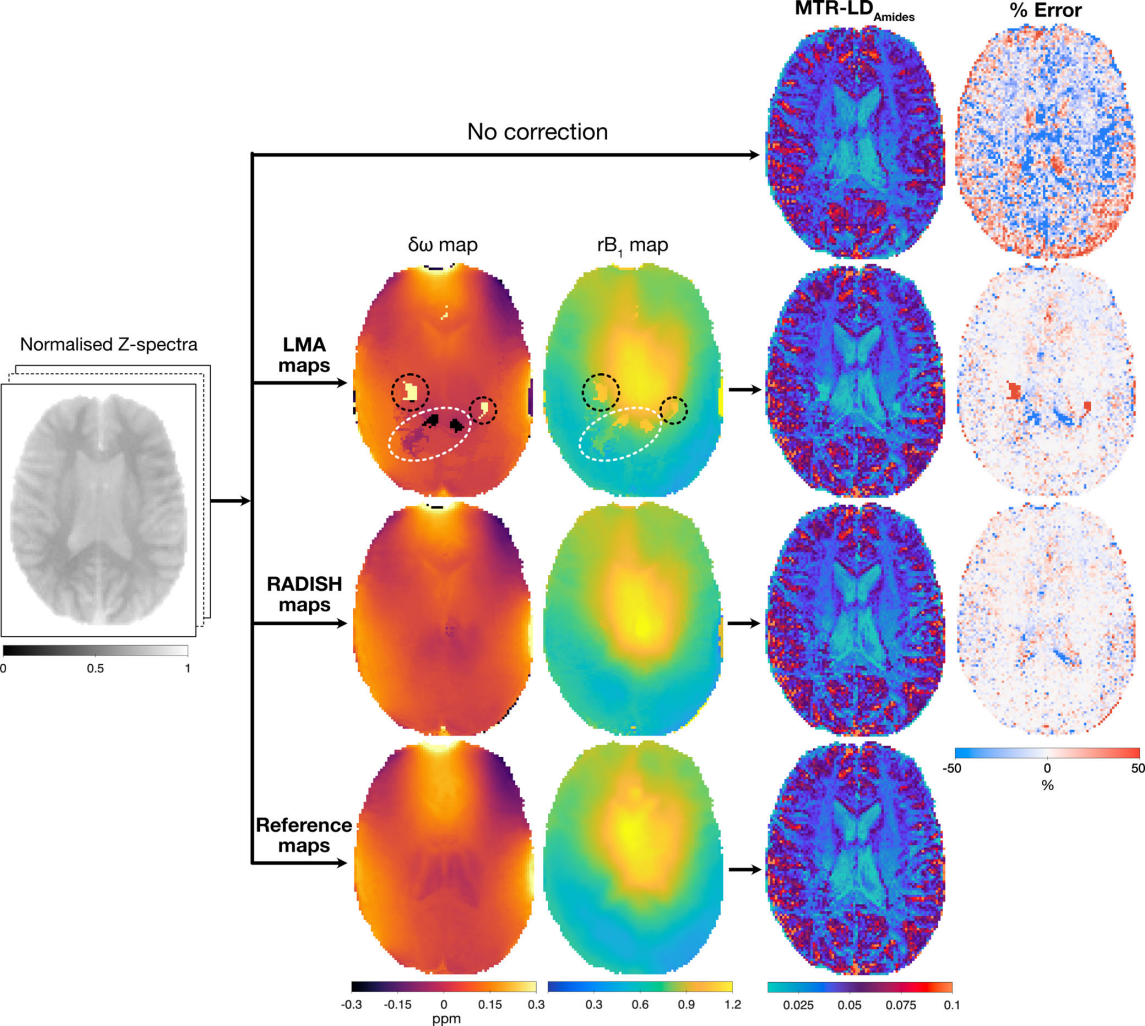
Amide‐CEST contrast before (top row) and after δω and $B_{1}$ correction using WASABI‐LMA (second row) and WASABI‐RADISH (third row) maps, in comparison to contrast after correcting with reference maps (bottom row). Artifacted regions in LMA maps lead to both obvious erroneous regions (black circles) and hard to discern erroneous regions (white oval) in the final CEST map. Reference maps were WASSR and AFI for δω and $B_{1}$, respectively. ppm: parts per million, MTR‐LD Amide: Amide‐weighted contrast calculated from magnetization transfer ratio linear difference method.

Figure 6 provides an example of applying RADISH maps to correct for δω and $B_{1}$ inhomogeneities in an Amide‐weighted CEST image, using linear interpolation for δω correction and two‐point CEST‐contrast correction^22^ for $B_{1}$. The most significant improvement in CEST contrast post‐correction was observed in the posterior and peripheral regions (where r$B_{1}$ <50%). This improvement was seen in both RADISH‐corrected and LMA‐corrected CEST images. Higher relative differences were observed in both LMA‐ and RADISH‐corrected CEST images in some regions within the ventricular area. As expected, artifacted regions in LMA maps correlated to some obvious erroneous regions that also appeared as stand‐out patches in the final CEST image. However, some erroneous regions were harder to discern in the final image despite visible artifacts in the LMA maps (Figure 6, white oval).

### 3.5 Robustness at reduced sampling

Robustness was maintained when Z‐spectra were downsampled to 25 offsets from −1.5 to 1.5 ppm. Bland‐Altman results are shown in Table 2. Voxel‐pair spreads are similar to those between fully sampled RADISH and reference maps. Notably, the percentage of artifacted voxels was 0.96% across the head, lower than for the fully sampled spectra (1.04%), albeit minimally different.

**TABLE 2:** Bland‐Altman results for RADISH (49 offsets), downsampled RADISH (25 offsets) and LMA (49 offsets), with reference to each other and WASSR and AFI.

			Whole brain			Middle slices (5–8)		
			Includes *all* artifacted voxels in LMA maps			Includes *fewer* artifacted voxels in LMA maps		
	Method 1	Method 2	Bias	Lower	Upper	Bias	Lower	Upper
δω [ppm]	LMA	WASSR	0.011	−0.194	0.216	0.010	−0.062	0.082
	RADISH	WASSR	0.008	−0.075	0.092	0.010	−0.037	0.057
	Downsampled RADISH	WASSR	0.009	−0.077	0.095	0.010	−0.035	0.055
	RADISH	LMA	−0.003	−0.210	0.203	0.0001	−0.070	0.070
	Downsampled RADISH	LMA	−0.003	−0.210	0.204	0.0002	−0.067	0.067
r$B_{1}$ [au]	LMA	AFI	−0.007	−0.181	0.167	−0.037	−0.123	0.048
	RADISH	AFI	−0.009	−0.153	0.134	−0.023	−0.113	0.067
	Downsampled RADISH	AFI	−0.013	−0.156	0.130	−0.027	−0.113	0.060
	RADISH	LMA	−0.004	−0.156	0.149	0.015	−0.058	0.088
	Downsampled RADISH	LMA	−0.006	−0.161	0.148	0.013	−0.059	0.086

*Note*: Whole brain results refer to analyses performed on all 12 slices, which include large regions of artifacted voxels in LMA maps. Middle slices include the middle 4 slices, for which artifacted voxels in LMA make up less than 1% of the area. Across the whole brain, RADISH resulted in narrower limits of agreement (LoA) with reference techniques compared to LMA. The LoA between RADISH/downsampled RADISH and LMA across 12 slices was similar to that between LMA and reference techniques. However, as expected, once most artifacted voxels in the LMA maps were removed from statistical calculations, the LoA between LMA and reference techniques improved, and likewise between LMA and RADISH/downsampled RADISH.

Abbreviations: au, arbitrary unit; ppm, parts per million; r$B_{1}$, relative $B_{1}$; δω: Water shift.

### 3.6 Speed

The average processing speed per voxel was 9.28$\times 10^{-4}$ s using RADISH, compared to 0.181 s using the original approach; the proposed RADISH method is more than two orders of magnitude faster. In practical terms, eight average slices comprising 56 000 non‐zero voxels can be processed in under 7 s, compared to the original method requiring 21 min, when using eight parallel workers.

## 4 DISCUSSION

In this study, an alternative method has been proposed to derive water shift and excitation inhomogeneity maps from WASABI magnitude data. Comparisons were performed on 7 *in vivo* data sets (≈560 000 total voxels). Our proposed method, RADISH, has been shown to improve robustness across all brain slices with the chosen 3D‐GRE readout, with the most noticeable improvements seen toward the extreme ends of the head. This is particularly useful should a 3D imaging readout be chosen. The efficacy of increased accuracy in maps provided by RADISH in contrast to LMA was also demonstrated with an example of δω‐ and $B_{1}$‐corrected Amide‐CEST maps, where both visually distinctive (i.e., easy to detect in quality control) and non‐apparent confounding contrasts were mitigated. There are several explanations for why extreme head regions are challenging for the original LMA‐based approach. Firstly, these regions are more prone to noise and motion artifacts, resulting in lower SNR. Secondly, it has previously been discussed^19^ that the discontinuities in WASABI curves, caused by depolarization in the magnitude data, increase the fitting complexity and the risk of convergence to local minima. Finally, the LMA approach requires a look‐up step prior to fitting, where each raw WASABI curve is compared to a static collection of 36,792 possible curves to find the most probable starting parameters.^17^, ^21^ The rigidity of this collection, combined with LMA's reliance on starting parameters for fit efficacy, means that regions with $T_{1}$ or $T_{2}$ decay or initial magnetization outside the predicted range will fail to produce sensible results. While in theory the user can introduce their own ranges of parameters to increase the number of candidate starting parameters, this would significantly increase post‐processing time. In addition, efficiency in testing and selecting these ranges would be impeded by the slow processing speed. RADISH, conversely, lessens the overall optimization complexity, relying solely on the locations of the curve's maxima; confounds introduced by factors such as motion and noise are greatly reduced. This ensures recovery of information even in the presence of head motion in more than one direction, in a few offsets.

Z‐spectral asymmetry is an additional phenomenon that can be challenging for LMA. In *in vivo* data, these asymmetries are likely a combination of imaging artifacts, noise, and head motion. As the WASABI model (Equation (1)) assumes symmetry, the efficacy of a nonlinear least square fit is reliant on the same. One can reduce this by increasing the magnetization recovery time ($t_{rec}$) or acquiring more than one $M_{0}$ image. However, both of these interventions increase acquisition time, which then risks head motion. In contrast, RADISH's use of maxima locations to generate initial starting parameters and Z‐spectra derivatives as the objective function allows the reliable retrieval of δω and $B_{1}$ values in asymmetrical spectra, providing robustness in sub‐optimal acquisition conditions (e.g., voxel as seen in Figure 2 See Supplementary Material Section S2 for more example).

Our results showed that RADISH produced δω and r$B_{1}$ maps in strong agreement with LMA maps, within a fraction of the required computational time. Using WASSR to generate reference δω maps suggested that RADISH could provide maps with precision on par with a “gold‐standard” technique, with artifacts limited to ≈1% of all areas.

Outside artifacted regions, we observed the greatest divergence between RADISH δω and WASSR in the extreme inferior and superior slices, particularly just anterior to the genu. This region is prone to large δω shifts due to its proximity to the nasal cavity, which can amplify artifacts in spectra. However, these differences are also seen when comparing LMA with WASSR, suggesting that the divergence stemmed from the raw Z‐spectra and therefore the acquisition methods directly. Furthermore, the qualities of WASSR maps are still dependent on SNR, the fitting method, and the algorithm of choice,^5^, ^23^, ^24^ and may differ from one scanning site to another.

For $B_{1}$ maps, the largest differences between RADISH and AFI are in the posterior region of the inferior slices. This may be due to the low r$B_{1}$ in these regions (< 40%) affecting the SNR of either WASABI or AFI images, or both. However, the use of AFI maps in this study was strictly to provide comparative results between a common $B_{1}$/flip angle mapping technique and WASABI, in addition to identifying potential outlier regions in WASABI $B_{1}$ maps. Drawbacks of AFI, including requirements for interpolation and probable distortion by $B_{0}$ inhomogeneity,^25^ particularly with low SNR,^26^ prevent it from being considered “ground truth”. WASABI as a $B_{1}$ mapping technique has been previously compared against Bloch‐Siegert Shift mapping, showing a mean difference of −0.0187 for r$B_{1}$ at 7T.^17^ As such, we refrained from inferring conclusions regarding accuracy based on voxel‐wise comparison with AFI and were concerned solely with validating RADISH against LMA.

One key limitation in the use of WASABI and WASSR as $B_{0}$ mapping techniques is the relatively long acquisition times compared to other methods, due to the need for finely‐sampled frequency offsets. As reported, the number of acquired WASABI offsets can be reduced to 21 while still achieving similar levels of accuracy.^17^ Likewise, our analysis showed that RADISH maintained robustness at 25 offsets. Time can be further saved with a reduction in offset number and use of parallel imaging; however, this would still be incompatible with applications such as shimming, pTx pulse optimization, or inter‐offset δω correction, where immediacy is a top priority. However, use of WASABI & RADISH is compelling when the experiments require high precision in δω and $B_{1}$ maps, low SAR, insensitivity to $T_{1}$, δ $B_{0}$, and off‐resonance effects, and/or maximal consistency with CEST images with respect to FOV, matrix size, and field distortions. One further advantage worth noting is that WASABI & RADISH also provides a frequency map free of wrapping. These requirements are typically expected for CEST studies and could be expanded for use in distortion correction for EPI‐based sequences. The acceleration provided by RADISH will also benefit dynamic $B_{0}$ mapping using WASABI, such as in the case of $B_{0}$ drift correction in gagCEST.^27^ Finally, RADISH may also be used with the recently introduced WASABITI method^18^ to simultaneously provide δω, $B_{1}$, and $T_{1}$ maps.

The main complexity that remains in the RADISH algorithm is the steps to find the optimal pair of maxima for each voxel. Given the diversity of shapes that WASABI Z‐spectra can take on in combination with noise and incomplete recovery or saturation, it is not possible to use an algorithm based on symmetry alone to detect δω, as one can for WASSR. An algorithm or protocol that could reliably detect the axis of symmetry in Z‐spectra would further significantly speed up post‐processing time via vectorization and may be worthy of investigation. One candidate may be the acquisition of a frequency map with a fast, albeit imprecise, sequence (e.g., FLASH) to use as a guide towards the “true” δω. Alternatively, one could acquire CEST images first and use the global minima in the CEST Z‐spectra as starting points to search for the precise δω and subsequent $B_{1}$ values. The analytical solution from this work provides a starting point for your creativity indulgence.

## 5 CONCLUSION

The proposed method, RADISH, provides increased robustness and significant post‐processing acceleration for WASABI δω and r$B_{1}$ mapping, achieving multi‐slice whole brain results within seconds. Improved robustness was demonstrated, particularly at the extremes of the head, with an artifact reduction of up to 15.06%. This ensures reliable generation of 3D δω and $B_{1}$ maps using WASABI as an acquisition method, even when acquisition parameters are not optimized for all regions of the brain. These improvements contribute to making quantitative CEST imaging at ultra‐high‐field more reliable and feasible for clinical implementation.

## CONFLICT OF INTEREST STATEMENT

The authors declare no potential conflict of interests.

## FUNDING INFORMATION

This work was supported by the Melbourne Research Scholarship, a Graeme Clark Institute Top‐up Scholarship, and partially supported by the National Health and Medical Research Council grant 1174040.

## Supporting information


**Data S1**: **Figure S1.** Two examples of WASABI curves with extreme asymmetry that do not affect the performance of RADISH. (A) An interior tissue boundary voxel with asymmetry at ≈0.2 ppm, which causes reduced trough prominence. RADISH is able to return δω and B1 values similar to references, while LMA converges to local minima. Some asymmetry is also observed in the raw WASSR curve. (B) A tissue boundary voxel with extreme asymmetry at ≈0 ppm, which causes the trough to diminish to the extent that it forms a saddle point. Both RADISH and LMA are able to return δω and rB1 close to reference values. The reference mapping techniques were WASSR and AFI for δω and r$B_{1}$, respectively. s: seconds, ppm: parts per million. **Figure S2.** $B_{1}$ and δω maps generated by WASABI & LMA (left column, original method), WASABI & RADISH (middle column, proposed method), and reference techniques (right column). The reference technique for $B_{1}$ and δω mapping was AFI and WASSR, respectively. au: arbitrary unit, ppm: parts per million. **Figure S3.** Example of a Z‐spectrum within the lateral ventricle. Yellow boxes indicate the best‐scoring curve returned by RADISH based on the choice of objective function.

## Data Availability

The data that supports the findings of this study are available from the corresponding author upon reasonable request. RADISH is available for access via https://github.com/maraquach/radish.

## APPENDIX A MAXIMA DISTANCE AND $B_{1}$ RELATIONSHIP

Isolating the second sine term in Equation (1) gives a function with the argument Δω and the decision variables δω and $B_{1}$:

(A1)
$$g_{0}(\Delta \omega )=\sin^{2}\left(\pi \;t_{p}\;\sqrt{{\left(\bar{\gamma }\;B_{1}\right)}^{2}+{\left(\Delta \omega -\delta \omega \right)}^{2}}\right)$$

Utilizing its symmetric nature, the model is simplified further by assuming no water shift (δω=0):

(A2)
$$g(\Delta \omega )=\sin^{2}\left(\pi \;t_{p}\;\sqrt{{\left(\bar{\gamma }\;B_{1}\right)}^{2}+{\left(\Delta \omega \right)}^{2}}\right)$$

Using the chain rule:

(A3)
$$g^{\prime }(\Delta \omega )=\frac{\pi \;t_{p}\;\Delta \omega \;\sin(t_{p}\;\sqrt{2\pi \;{\left(\bar{\gamma }\;B_{1}\right)}^{2}+{\left(\Delta \omega \right)}^{2}}}{\sqrt{{\left(\bar{\gamma }\;B_{1}\right)}^{2}+{\left(\Delta \omega \right)}^{2}}}$$

Maxima locations are found by solving for non‐zero Δω when $g^{\prime }(\Delta \omega )=0$. The roots of Equation (A3) occur at

(A4)
$$\begin{array}{cccc} t_{p}\;\sqrt{{\left(\bar{\gamma }\;B_{1}\right)}^{2}+{\left(\Delta \omega \right)}^{2}} & =\frac{n}{2},\text{where }n\in \mathbb{Z},n\neq 0 & & \text{[Hz]}\\ ∴\Delta \omega & =\sqrt{{\left(\frac{n}{2\;\omega_{0}\;t_{p}}\right)}^{2}-{\left(\frac{\bar{\gamma }\;B_{1}}{\omega_{0}}\right)}^{2}} & & \text{[ppm]} \end{array}$$

where n=−2 and n=2 provide the locations of the positive and negative Z(Δω) maxima adjacent to the axis of symmetry (δω=0) (Figure 1), respectively, assuming Δω direction as per CEST convention. Since the model is symmetric, the distance between these maxima is therefore:

(A5)
$$\begin{array}{cccc} x & =2\;\Delta \omega_{g^{\prime }=0,n=2} & & \\ & =2\sqrt{{\left(\frac{1}{\omega_{0}\;t_{p}}\right)}^{2}-{\left(\frac{\bar{\gamma }\;B_{1}}{\omega_{0}}\right)}^{2}} & & \text{[ppm]}\;\text{for}\;B_{1}<\frac{1}{\bar{\gamma }\;t_{p}} \end{array}$$

This provides the final relationship between $B_{1}$ value and the maxima pair distance:

(A6)
$$B_{1}=\frac{\omega_{0}}{\bar{\gamma }}\;\sqrt{{\left(\frac{1}{\omega_{0}\;t_{p}}\right)}^{2}-\frac{x^{2}}{4}}\;[\mu T]\;\text{for}\;x\leq \frac{2}{\omega_{0}\;t_{p}}$$

For $B_{1}>\frac{1}{t_{p}\;\bar{\gamma }}$, the same relationship as above is solved using n=4 (the second even n):

(A7)
$$B_{1}=\frac{\omega_{0}}{\bar{\gamma }}\;\sqrt{{\left(\frac{2}{\omega_{0}\;t_{p}}\right)}^{2}-\frac{x^{2}}{4}}\;[\mu T]\;\text{for}\;x\leq \frac{4}{\omega_{0}\;t_{p}}$$

Assuming that the measured $B_{1}$ does not exceed $\frac{2}{t_{p}\;\bar{\gamma }}$ (≈250% of nominal $B_{1}$ of 3.7 μT), there exists one unique $B_{1}$ for every maxima‐pair distance x. Thus, the following generalization is true for $x<\frac{4}{\omega_{0}\;t_{p}}$:

(A8)
$$B_{1}=\frac{\omega_{0}}{\bar{\gamma }}\;\sqrt{{\left(\frac{n}{\omega_{0}\;t_{p}}\right)}^{2}-\frac{x^{2}}{4}},\text{where}\;n=\left\{\begin{array}{l} 1,\;\text{if}\;x\leq \frac{2}{\omega_{0}\;t_{p}}\\ 2,\;\text{otherwise} \end{array}\right.$$

The relationship between $B_{1}$ and the maxima pair distance is graphically visualized in Figure A1. To ensure robust mapping in voxels where $x\approx \frac{2}{\omega_{0}t_{p}}$ (≈1.3 ppm in Figure A1), the optimization steps in RADISH use x instead of $B_{1}$, and precision can be controlled by changing adjustment factors $l_{1}$ and $l_{2}$ in Step 6 (Methods Section 2.2).
