# Supplementary material for: Rapid and simplified post‐processing for simultaneous B0 and B1 mapping in the application of CEST
Source: Magn Reson Med. 2025 Jul 22;94(6):2702–14. doi: 10.1002/mrm.70001 (PMC12501747; doi:10.1002/mrm.70001)
Supplement: Supplementary file 1 — Data S1: Figure S1. Two examples of WASABI curves with extreme asymmetry that do not affect the performance of RADISH. (A) An interior tissue boundary voxel with asymmetry at ≈0.2 ppm, which causes reduced trough prominence. RADISH is able to return δω and B1 values similar to references, while LMA converges to local minima. Some asymmetry is also observed in the raw WASSR curve. (B) A tissue boundary voxel with extreme asymmetry at ≈0 ppm, which causes the trough to diminish to the extent that it forms a saddle point. Both RADISH and LMA are able to return δω and rB1 close to reference values. The reference mapping techniques were WASSR and AFI for δω and rB1, respectively. s: seconds, ppm: parts per million. Figure S2. B1 and δω maps generated by WASABI & LMA (left column, original method), WASABI & RADISH (middle column, proposed method), and reference techniques (right column). The reference technique for B1 and δω mapping was AFI and WASSR, respectively. au: arbitrary unit, ppm: parts per million. Figure S3. Example of a Z‐spectrum within the lateral ventricle. Yellow boxes indicate the best‐scoring curve returned by RADISH based on the choice of objective function. [file MRM-94-2702-s001.pdf]

Supplementary material for

Rapid and simplified post-processing for simultaneous B_0 and B_1 mapping in the application of CEST

Mara Quach[1,2], Myrte Strik[2,3,4], Rebecca Glarin[2], Bradford A. Moffat[2], David K. Wright[5], Leigh A. Johnston[1,2]

[1] *Department of Biomedical Engineering and Graeme Clark Institute, The University of Melbourne, Parkville, Australia*
[2] *Melbourne Brain Centre Imaging Unit, The University of Melbourne, Parkville, Australia*
[3] *Spinoza Centre for Neuroimaging, Amsterdam, Netherlands*
[4] *Department of Computational Cognitive Neuroscience & Neuroimaging, Netherlands Institute for Neuroscience, Royal Netherlands Academy of Sciences (KNAW), Amsterdam, Netherlands*
[5] *Department of Neuroscience, The School of Translational Medicine, Monash University, Melbourne, Australia*

Corresponding author: Mara Quach
https://orcid.org/0000-0001-5158-6724

Key abbreviations

WASABI: Water Shift and B_1
RADISH: Rabi Distance Search
LMA: Levenberg-Marquardt Algorithm
WASSR: Water Shift Saturation Referencing
AFI: Actual Flip Angle

S1. Asymmetry

While the WASABI model predicts an even and symmetrical Z-spectrum, in *in vivo* data, amplitude asymmetry is not uncommon in regions containing tissue boundaries, particularly in the superior and inferior slices. These asymmetries are likely a combination of imaging artifacts, noise, and head motion.

It is key to the RADISH algorithm that asymmetry does not confound the generation of maps. The use of maxima locations to generate initial starting parameters and cases considered in Step 3 (Methods, Section 2.2) is robust against asymmetry. Fig S1A shows an example tissue boundary voxel with significant asymmetry that reduces the trough depth at ~0.2 ppm which leads to artifact in the raw WASABI curve. However, accurate $\delta\omega$ and B_1 are still detected by RADISH (difference of 3.2 Hz for $\delta\omega$ and 0.38 % for rB_1 compared to references). In the majority of cases, the amplitude asymmetry does not affect the algorithm's determination of maxima if a peak-finding algorithm that relies on the zero-crossing of the derivative curve is used in Step 2. In the rare case that extreme asymmetry results in the loss of a detectable peak, the three different Cases considered as outlined in Step 3 are enough to overcome this issue. An example of such an extreme scenario is presented in Fig. S1B at a boundary voxel, where the trough at ~0 ppm is diminished due to asymmetry to the extent that it forms a saddle point. The resulting accurate $\delta\omega$ and B_1 are still detected (difference of 4.5 Hz for $\delta\omega$ and 1.4 % for rB_1 compared to references).

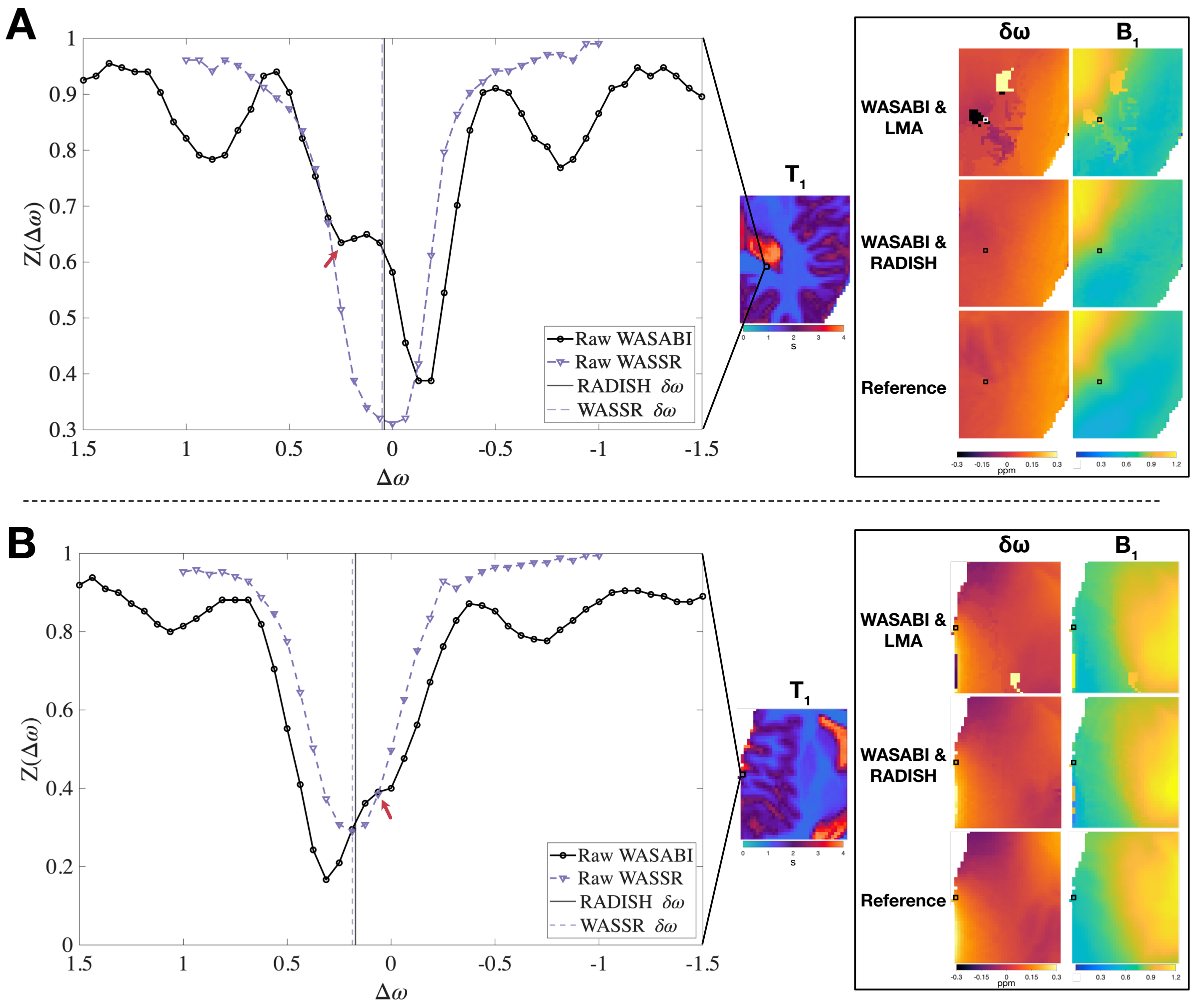

Figure S1. Two examples of WASABI curves with extreme asymmetry that does not affect the performance of RADISH. A) An interior tissue boundary voxel with asymmetry at ~0.2 ppm which causes reduced trough prominence. RADISH is able to return δω and B_1 values similar to references, while LMA converges to local minima. Some asymmetry is also observed in the raw WASSR curve. B) A tissue boundary voxel with extreme asymmetry at ~0 ppm which causes the trough to diminish to the extent that it forms a saddle point. Both RADISH and LMA are able to return δω and rB_1 close to reference values. The reference mapping techniques were WASSR and AFI for δω and rB1, respectively. s: seconds, ppm: parts per million.

S2. Qualitative comparison

Fig. S2 provides the full 12 slices from the same subject as shown in Fig. 3 in the main text.

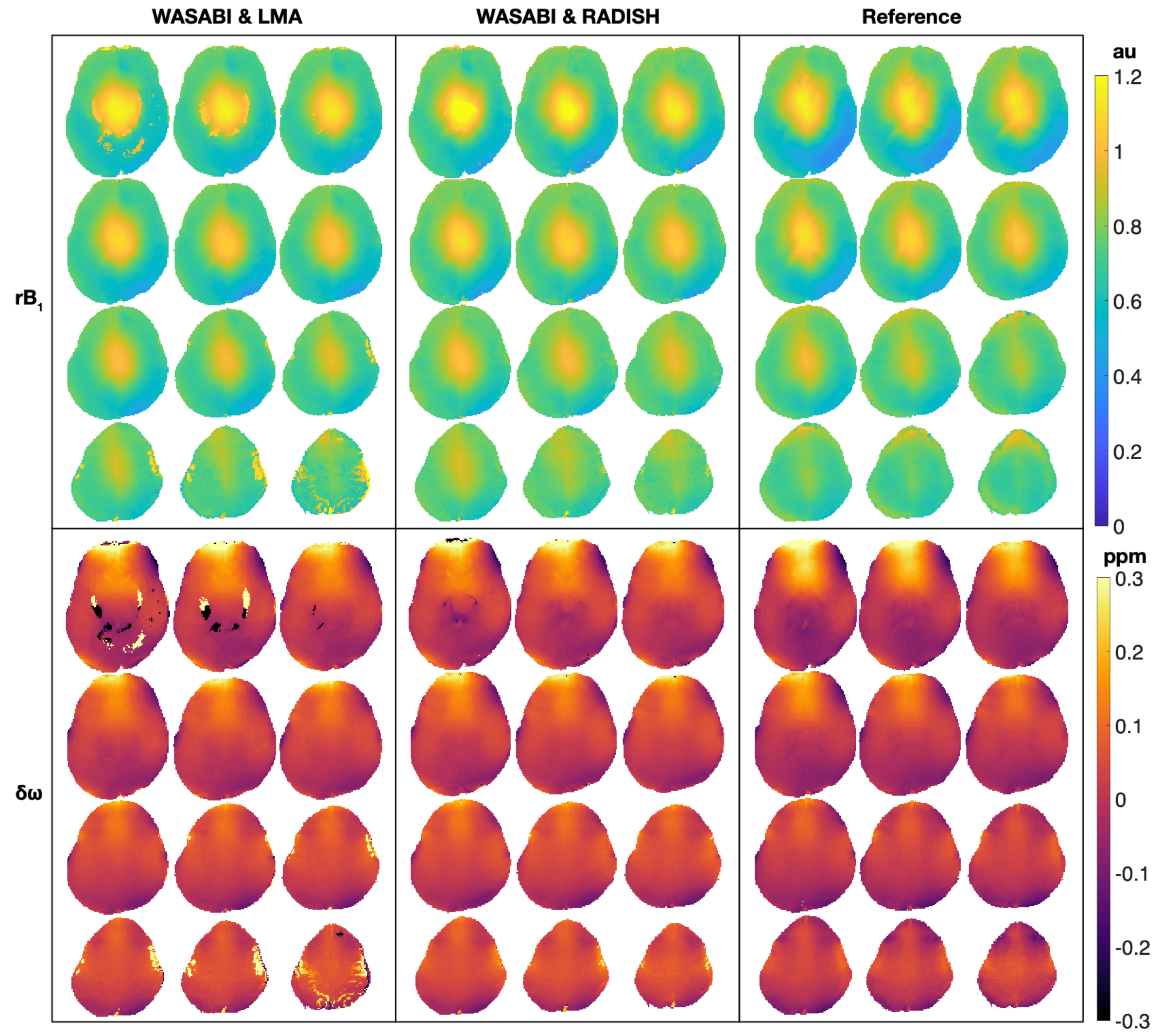

Figure S2. B_1 and δω maps generated by WASABI & LMA (left column, original method), WASABI & RADISH (middle column, proposed method), and reference techniques (right column). The reference technique for B_1 and δω mapping was AFI and WASSR, respectively. au: arbitrary unit, ppm: parts per million.

S3. Derivatives as objective function

It is crucial that the derivatives of the observed and candidate Z-spectra are used as the objective function. Amplitude variations in Z-spectra are majorly influenced by relaxations which are modelled, albeit incompletely, by parameters c and d in the original model by Schuenke *et al.* In using the derivative, the number of parameters to optimise is reduced to three – as c is a linear term. The benefits are greatly realised in regions with long T_1 (e.g., the lateral ventricle) where there is more likely to be incomplete longitudinal recovery in-between offsets i.e., $c << 1$. An example is provided in Fig. S3. Here, two candidate curves identified by RADISH in steps 1 – 4 are presented. While the first curve was constructed with the B_1 and δω closer to the correct values, it is penalised by its baseline amplitude. However, this is rectified when the derivative is considered for the objective function. The slice result also demonstrates overall improvement to δω and especially B_1 maps when using derivatives.

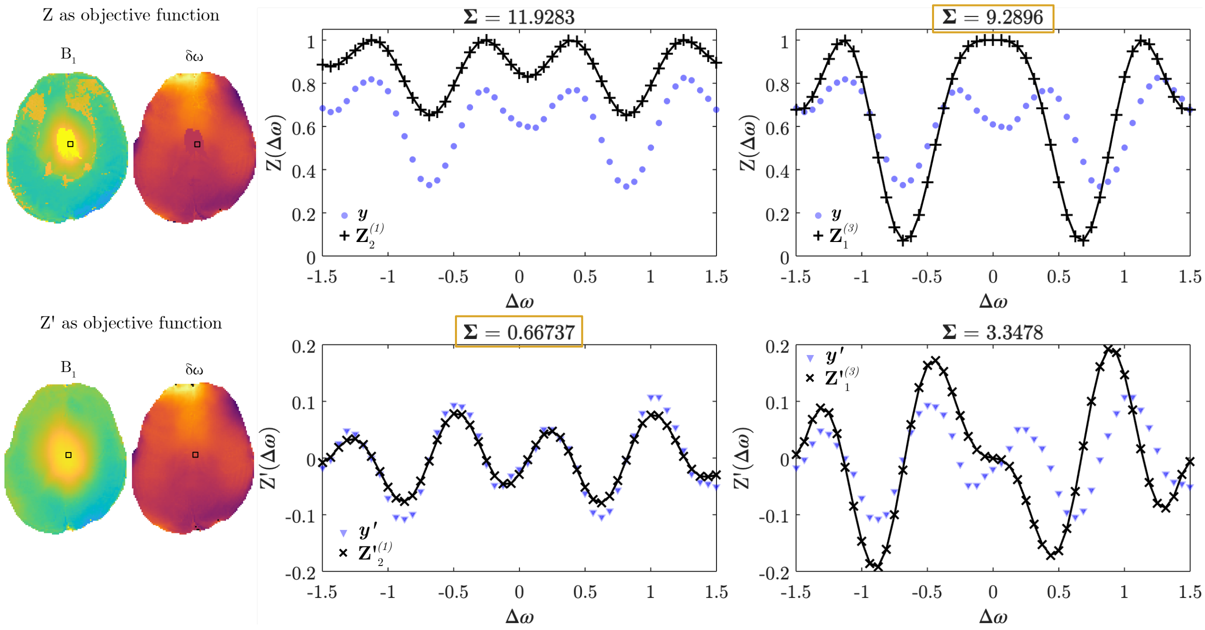

Figure S3. Example of a Z-spectrum within the lateral ventricle. Yellow boxes indicate the best scoring curve returned by RADISH based on the choice of objective function.
